# Genes suppressed by DNA methylation in non-small cell lung cancer reveal the epigenetics of epithelial–mesenchymal transition

**DOI:** 10.1186/1471-2164-15-1079

**Published:** 2014-12-08

**Authors:** Steven H Lin, Jing Wang, Pierre Saintigny, Chia-Chin Wu, Uma Giri, Jing Zhang, Toshi Menju, Lixia Diao, Lauren Byers, John N Weinstein, Kevin R Coombes, Luc Girard, Ritsuko Komaki, Ignacio I Wistuba, Hiroshi Date, John D Minna, John V Heymach

**Affiliations:** Department of Radiation Oncology, The University of Texas MD Anderson Cancer Center, 1515 Holcombe Blvd., Unit 097, Houston, TX 77030 USA; Department of Bioinformatics and Computational Biology, The University of Texas MD Anderson Cancer Center, 1515 Holcombe Blvd., Unit 097, Houston, TX 77030 USA; Cancer Research Center of Lyon, UMR INSERM 1052 / CNRS 5286 Mixte CLB, Centre Léon Bérard, 28 rue Laennec, 69373 Lyon, Cedex 08, France; Department of Genomic Medicine, The University of Texas MD Anderson Cancer Center, Houston, Texas USA; Department of Thoracic/Head and Neck Medical Oncology, The University of Texas MD Anderson Cancer Center, 1515 Holcombe Blvd., Unit 097, Houston, TX 77030 USA; Ohio State University College of Medicine, 250 Lincoln Tower, 1800 Cannon Drive, Columbus, Ohio USA; Hamon Center for Therapeutic Oncology, The University of Texas Southwestern Medical Center, Dallas, Texas USA; Department of Pathology, The University of Texas MD Anderson Cancer Center, Houston, Texas USA; Department of Thoracic Surgery, Kyoto University Hospital, Kyoto, Japan

**Keywords:** DNA methylation, Epithelial-mesenchymal transition, Erlotinib, Lung cancer

## Abstract

**Background:**

DNA methylation is associated with aberrant gene expression in cancer, and has been shown to correlate with therapeutic response and disease prognosis in some types of cancer. We sought to investigate the biological significance of DNA methylation in lung cancer.

**Results:**

We integrated the gene expression profiles and data of gene promoter methylation for a large panel of non-small cell lung cancer cell lines, and identified 578 candidate genes with expression levels that were inversely correlated to the degree of DNA methylation. We found these candidate genes to be differentially methylated in normal lung tissue versus non-small cell lung cancer tumors, and segregated by histologic and tumor subtypes. We used gene set enrichment analysis of the genes ranked by the degree of correlation between gene expression and DNA methylation to identify gene sets involved in cellular migration and metastasis. Our unsupervised hierarchical clustering of the candidate genes segregated cell lines according to the epithelial-to-mesenchymal transition phenotype. Genes related to the epithelial-to-mesenchymal transition, such as *AXL, ESRP1, HoxB4*, and *SPINT1/2*, were among the nearly 20% of the candidate genes that were differentially methylated between epithelial and mesenchymal cells. Greater numbers of genes were methylated in the mesenchymal cells and their expressions were upregulated by 5-azacytidine treatment. Methylation of the candidate genes was associated with erlotinib resistance in wild-type *EGFR* cell lines. The expression profiles of the candidate genes were associated with 8-week disease control in patients with wild-type *EGFR* who had unresectable non-small cell lung cancer treated with erlotinib, but not in patients treated with sorafenib.

**Conclusions:**

Our results demonstrate that the underlying biology of genes regulated by DNA methylation may have predictive value in lung cancer that can be exploited therapeutically.

**Electronic supplementary material:**

The online version of this article (doi:10.1186/1471-2164-15-1079) contains supplementary material, which is available to authorized users.

## Background

The methylation of DNA is involved in the control of chromatin folding, protein complex assembly, and gene expression. It is a normal process of cellular development, aging, paternal/maternal genetic imprinting, and X-chromosome inactivation [1]. DNA methylation is the only known heritable modification of DNA, and is aberrant in cancer, involving both excessive and insufficient methylation of DNA regions of cytosine–guanine bonds (CpG), which then alters the expression of certain genes [2]. The aberrant hypermethylation of dense CpG sites in many cancers occurs at tumor suppressor genes in a non-random, tumor-specific pattern [3].

Given the possible role of aberrant DNA methylation in cancer initiation and progression, a significant effort has been directed to identify DNA methylation biomarkers in cancer and to use such markers to predict therapeutic responses and disease prognosis [4]. A notable example is the association between the silencing of the *O6-methylguanine-DNA methyltransferase* (*MGMT*) gene and the improved response to temozolomide and radiation in patients with glioblastoma [5]. Others include the association of the methylation of *PTEN*, *IGFBP-3*, and various DNA repair enzymes (hMLH1, MRN, BRCA1, ATM, and the *FANC* genes) with either resistance (*PTEN*, *IGFBP-3*) or sensitivity (DNA repair enzymes) to chemotherapy or radiation [6–10]. For lung cancer, DNA methylation of genes may be useful for assessing cancer risk based on the analysis of the sputum of smokers [11], and may be prognostic in early-stage lung cancer [12, 13].

To better understand the DNA methylation changes associated with gene expression in lung cancer, we determined the methylation status of more than 27,000 CpG sites across 14,000 genes at selective promoter regions in 73 non-small cell lung cancer (NSCLC) cell lines using the Illumina HumanMethylation27 BeadChip. We integrated this dataset with the gene expression profiles of the same panel of cell lines to identify the genes with expression levels that were most affected by DNA promoter methylation. We sought to determine the biologic significance of genes that were significantly repressed in association with methylation in silico, as well as experimentally, both *in vitro* and *in vivo.*

## Results and discussion

### Identifying genes aberrantly associated with DNA methylation in NSCLC Cell Lines

**Figure 1:**
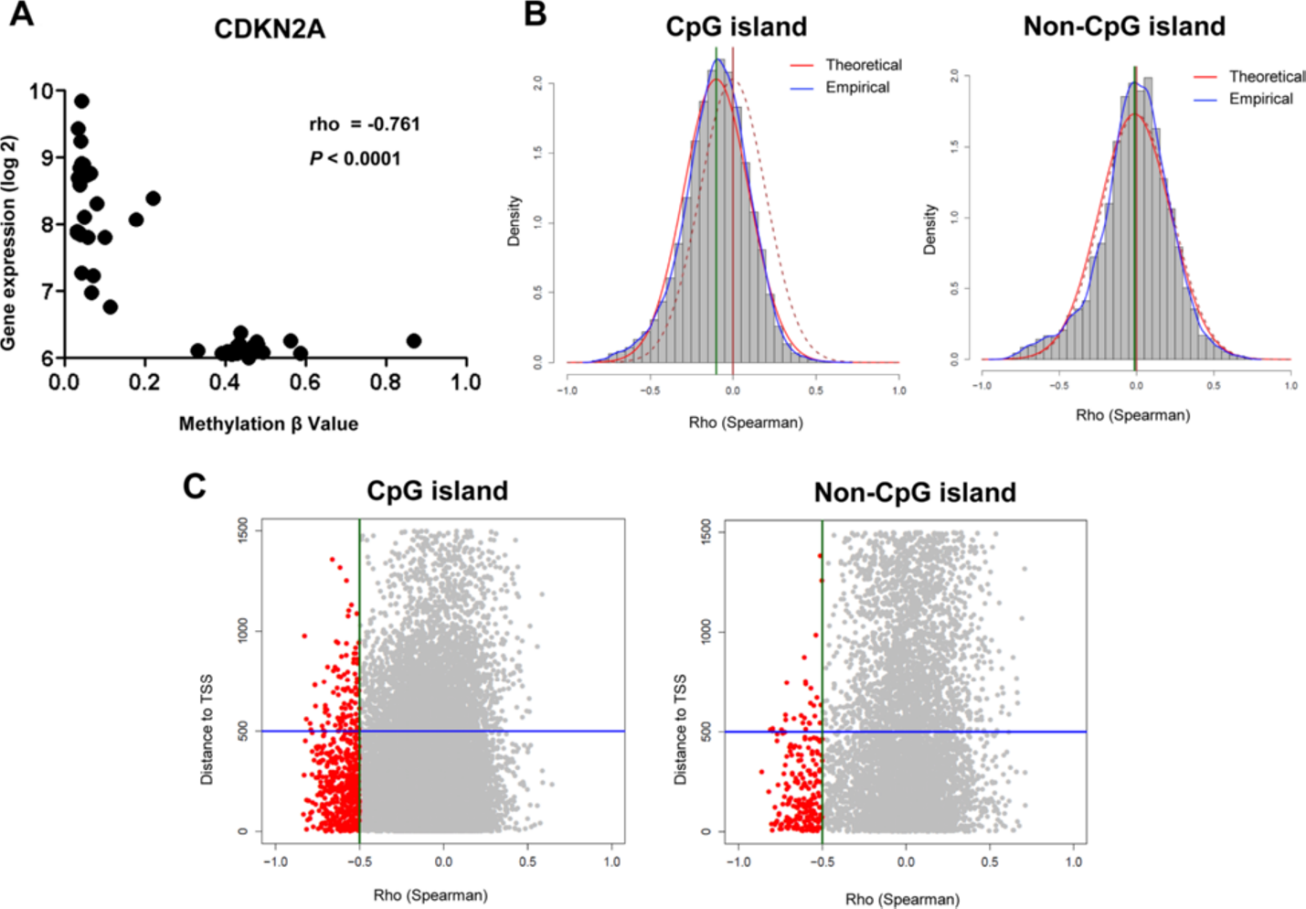
**Differential genes are significantly repressed in association with methylation (SRAMs) in NSCLC cell lines. A)** Example of the relationship between gene expression and methylation status for a single probe interrogating a single CpG site within the promoter region of *CDKN2A*. **B)** Frequency histogram of the Spearman rho values for all probes that correspond to either CpG or non-CpG island sites. Red curves are theoretical Gaussian representation of the distribution of rho given the number of probes for each gene set and the actual mean and standard deviation values; blue curves represent the empirical distribution from the data; dashed burgundy lines represent normal distribution curves for comparison; and the arrow identifies the point where the two curves cross and an excess of probes are seen with a rho ≤ -0.5. **C)** Relationship of rho to the distance from the transcriptional start site (TSS) for CpG and non-CpGi probes. Most of the probes (~80%) that are candidate SRAMs cluster within 500 bp of the TSS.

For each of the 73 NSCLC cell lines examined, we analyzed the relationship between the degree of methylation (beta value 0 to 1, with 1 being completed methylated) of a particular gene and the expression level of that gene. We sought to identify genes that were significantly repressed in association with DNA methylation, which we subsequently refer to as SRAMs, a term first used in the study of breast cancer [14]. We computed the Spearman correlation of the relationship between methylation and gene expression for all the cell lines, assuming that genes negatively regulated by DNA promoter methylation have a negative Spearman rho value. This approach would only identify differentially regulated genes and not genes that are fully methylated or unmethylated in these cell lines. We first tested our approach by looking at the Spearman correlation for *CDKN2A*, a gene well known to be frequently downregulated by promoter methylation in NSCLC [15]. In the Illumina assay, four probes assessed four CpG sites within the *CDKN2A* promoter. We found that *CDKN2A* was differentially methylated in the various cell lines, and the degree of methylation was significantly and inversely correlated with gene expression (Figure 1A). This relationship was preserved in each of the four probes examined (Additional file 1: Figure S1).

**Figure 2:**
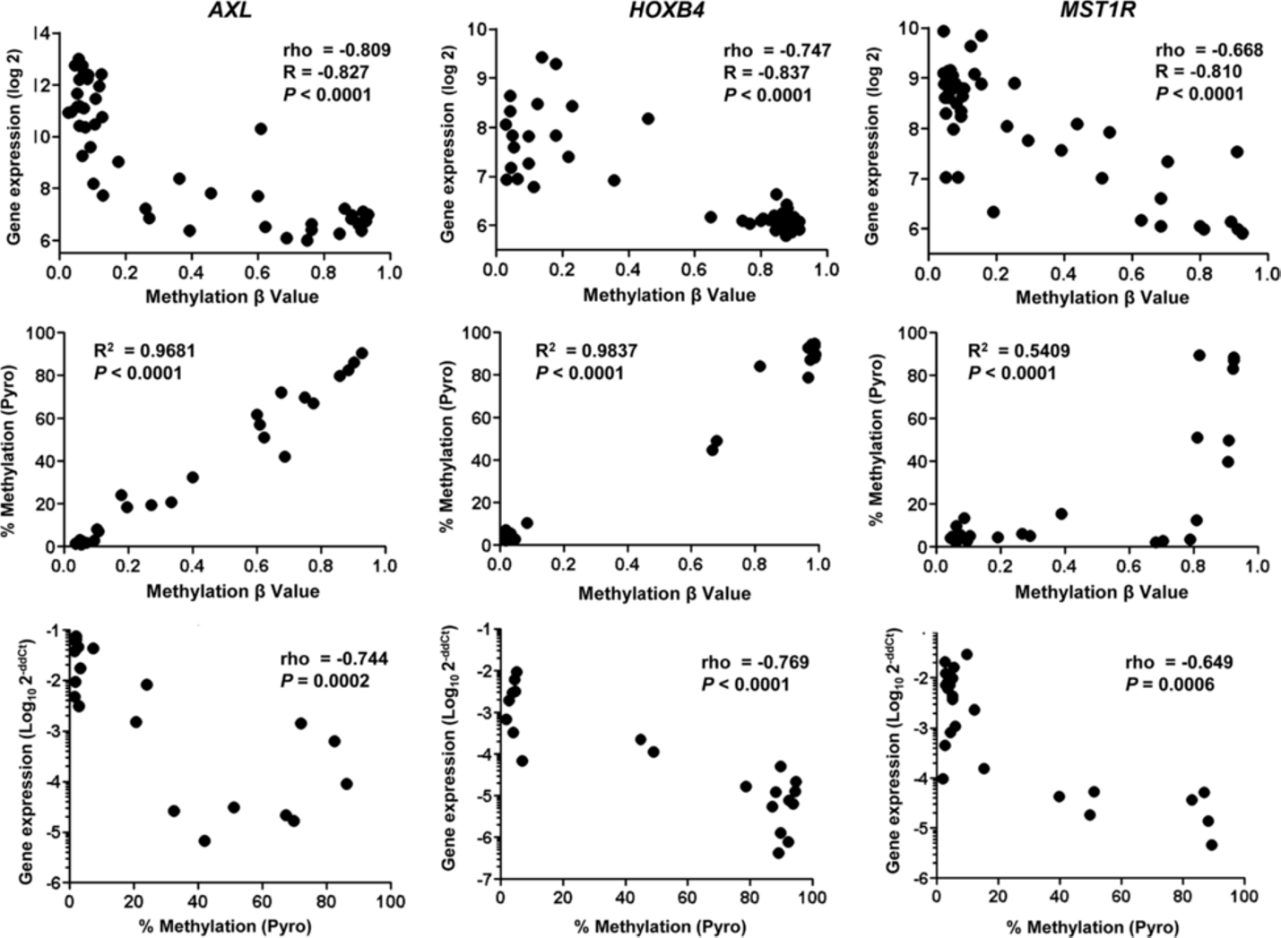
**Validation of the integrative analysis for select genes.** Three genes with CpG islands were evaluated. Top row is the relationship between array methylation data and Illumina gene expression; middle row provides Pearson correlation of the array methylation data with the pyrosequencing methylation status; and bottom row shows the relationship of methylation status by pyrosequencing with the quantitative real-time PCR results for the expression of these genes.

We expanded our analysis to the rest of the probes. We looked at the distribution of rho values for probes located within or outside of CpG islands (CpGi). Rho values of CpGi probes had a mean distribution that was significantly shifted to the left of 0 compared to that of non-CpGi probes (p < 0.00001, Figure 1B). For both sets of probes, an excess was seen in the experimental dataset at the rho cutoff of -0.5 that was more than expected when compared to a random distribution (p < 0.00001, Figure 1B). This was not observed for probes that had a positive correlation with gene expression. There were also no differences regarding whether the probes corresponded to CpGi or non-CpGi genes (3.3% vs. 3.5%, *P* = 0.48). Greater than 80% of these probes lie within 500 bp of the transcription starting site (TSS), corresponding to sites under transcriptional control by DNA methylation (Figure 1C). We found 750 probes that correspond to 578 unique SRAMs, genes with expression levels that were inversely correlated with the degree of DNA methylation (Additional file 2: Table S1). We found the same pattern as that of *CDKN2A* for other genes known to be regulated by methylation (*CDKN2B*, *MGMT*), as well as for many novel genes not previously known to be regulated by methylation (Additional file 3: Figure S2). We validated a set of SRAMs using pyrosequencing and quantitative real-time polymerase chain reaction (PCR) in 26 cell lines (Figure 2, Additional file 4: Figure S3).

### SRAMs differentiate tumor from normal lung and distinguish tumor subtypes

**Figure 3:**
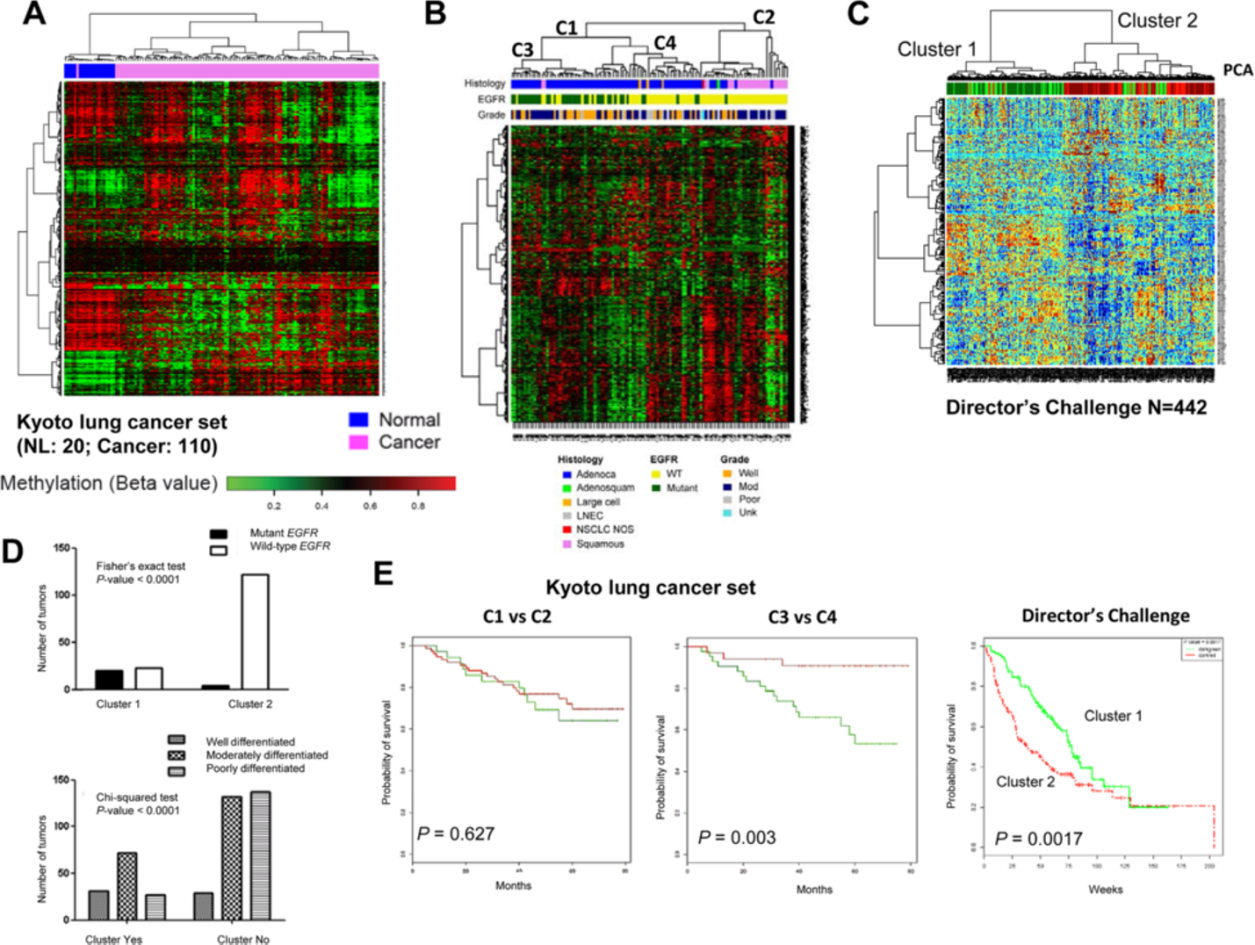
**NSCLC SRAMs are differentially methylated between normal and tumor tissues segregate into molecular pathologic subtypes. A)** Heatmap of the hierarchical clustering of 637 matched probes for SRAMs in the Kyoto set of 110 tumors and compared to 20 unmatched normal lung tissue samples. **B)** Using matched 637 probes from the HumanMethylation450 to the HumanMethylation27 platforms, unsupervised clustering of 110 tumors from the Kyoto tumor set was conducted, with tumor clustering along lines of histology (adenocarcinoma vs. squamous cell carcinoma), *EGFR* mutation status, and tumor grades. **C-D)** Unsupervised clustering based on gene expression of the matched gene regulatory module (GRM) genes on the Director’s Challenge dataset of 442 lung adenocarcinomas. The GRMs were able to segregate the tumors in the Director’s Challenge into two main groups, with *EGFR* mutations and well-to-moderately differentiated tumors in group 1, and wild-type *EGFR* and moderately-to-poorly differentiated tumors in group 2. **E)** SRAM segregates NSCLC tumors into prognostic groups in the Kyoto and Director’s Challenge tumor sets. Branch point C1 vs C2 mostly segregates squamous from adenocarcinoma, without a difference in prognosis; whereas branch point C3 vs. C4 is highly prognostic because of the separation of *EGFR* mutant from wild-type tumors. For the Director’s Challenge tumor set, the two clusters that preferentially separate *EGFR* mutant and wild-type tumors are also prognostic for survival.

As aberrant DNA methylation is one of the defining characteristics of cancer, SRAMs should reflect alterations that discriminate cancer from normal tissue and reflect the underlying tumor biology. We evaluated the methylation status of SRAMs in a set of 110 lung tumors from a Japanese cohort, hereafter termed the Kyoto tumor set, which included 20 unmatched normal lung tissues arrayed using the Illumina HumanMethylation450 BeadChip. Since the cell line SRAM dataset was generated from the HumanMethylation27 platform, we took 637 probes (520 unique genes) that overlapped between the two Illumina platforms for the analysis. We found that SRAMs discriminated NSCLC from normal lung tissue, with clusters of probes that were either predominantly methylated in normal tissue or methylated in tumors (Figure 3A). SRAMs also distinguished squamous carcinoma from adenocarcinoma, the two major histological subtypes of NSCLC, as well as lung adenocarcinomas with or without an *EGFR* mutation (Figure 3B). These observations were corroborated using SRAM expression profiles of lung adenocarcinomas included in the Director’s Challenge Consortium for the Molecular Classification of Lung Adenocarcinoma [16]. SRAMs preferentially separated *EGFR* mutants from wild-type tumors (Figure 3C and D). In the Kyoto tumor set, patients that separated into these clusters had significant survival differences, similar to that seen for the Director’s Challenge tumor set (Figure 3E).

### SRAMs and the epithelial-to-mesenchymal transition (EMT) phenotype

To further define the functional significance of genes negatively regulated by DNA promoter methylation, we performed a gene set enrichment analysis (GSEA) using the Spearman correlation rho value as the ranking variable [17]. The top gene sets that included genes negatively regulated by DNA promoter methylation had been obtained from diverse cancer datasets and were involved in various biologic functions, such as cell migration in bladder cancer and resistance to gefitinib in NSCLC, and included genes downregulated with E-cadherin knockdown in human breast mammary epithelial (HBME) cell lines, genes differentially expressed in metastatic melanoma, genes methylated in glioblastoma and pancreatic cancer, and genes differentially regulated in luminal vs. basal/mesenchymal breast cancer cells (Additional file 5: Table S2).

**Figure 4:**
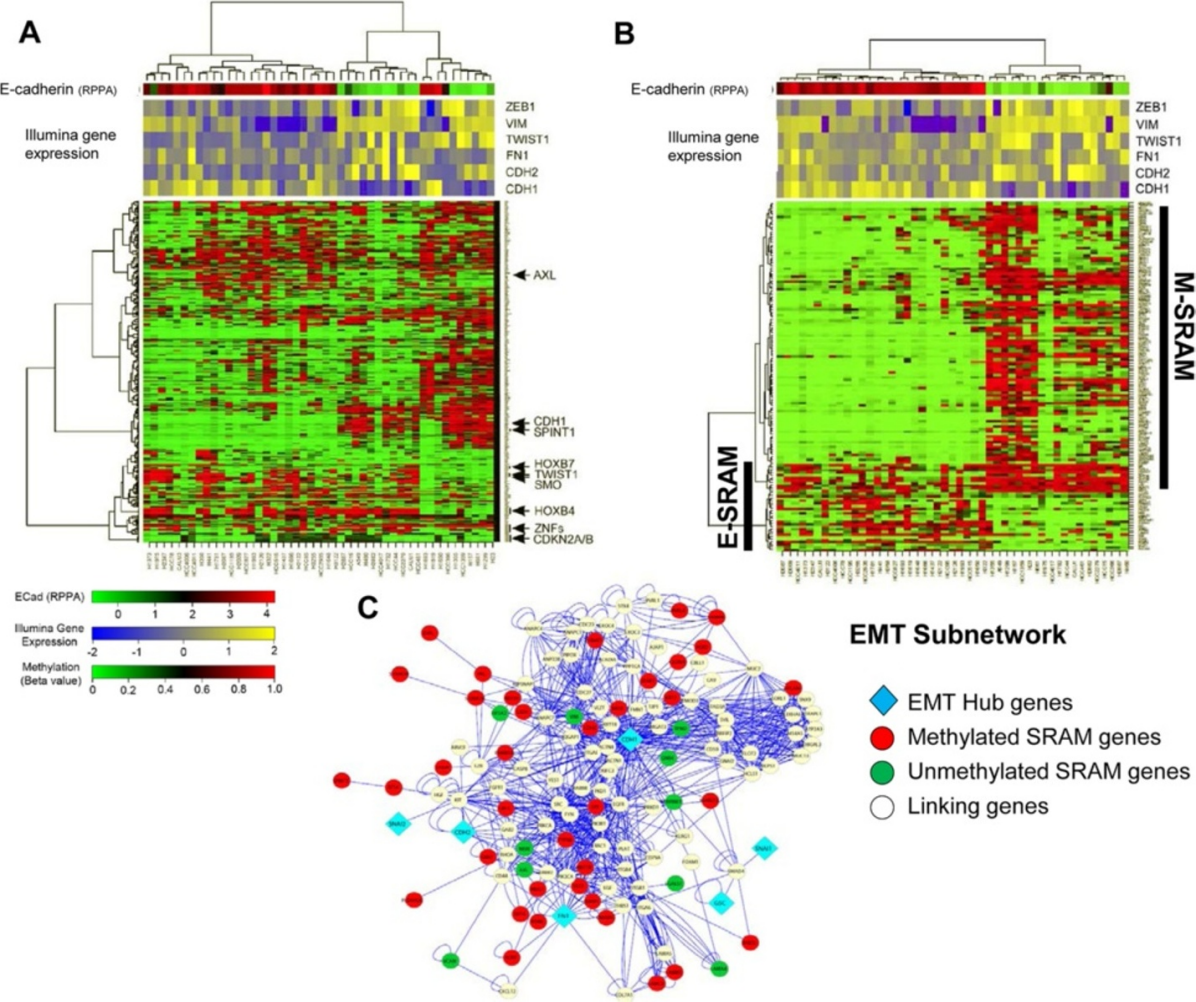
**Specific SRAM methylation patterns define epithelial (E) and mesenchymal (M) cell types. A)** Hierarchical clustering of SRAMs for a subset of cell lines (n = 47) with RPPA data for E-cadherin (ECAD) segregates cell lines into high and low ECAD groups. Representative genes that are preferentially unmethylated (*AXL*) or methylated (*CDH1*, *SPINT1*) in the mesenchymal cells are shown. Illumina gene expression levels of EMT markers (*ZEB1*, Vimentin (*VIM*), *Twist1*, Fibronectin (*FN1*), *CDH2*, and *CDH1*) are overlaid to confirm the E vs M identity of the cell lines. **B)** Wilcoxon rank sum test was used to identify the SRAMs that are differentially methylated between E and M cells, which we call the EMT-SRAMs. The identified 135 probes (111 unique genes) were used for hierarchical clustering of the cell lines. EMT-SRAMs that are predominantly methylated in M cells are called M-SRAMs, and those methylated in E cells are called E-SRAMs. **C)** EMT-SRAM association with EMT network genes using curated network analysis. Closest second neighbor analysis of genes associated with core EMT factors (“EMT Hub genes”: *CDH1, CDH2, FN1, SNAI1, SNAI2, GSC*) finds numerous associations with genes within the EMT-SRAM set. Red spheres represent SRAMs that are methylated in M cells; green spheres are those methylated in E cells; white spheres represent linking genes.

We postulated that biologic functions identified by the GSEA were related to the process of EMT, a developmental and adaptive cellular process that has been associated with resistance to cancer therapies and regulation of metastasis [18]. We performed a hierarchical cluster analysis of the cell lines using the SRAMs, and overlaid the relative protein expression of E-cadherin, a cell adhesion molecule that is downregulated during EMT and which plays a key role in the signaling and regulation of EMT [19], along with the expression of EMT-related genes *ZEB1, VIM, Twist1, FN1, CDH2,* and *CDH1*. We found that the identification of SRAMs clustered cells into two groups: “epithelial-like (E)” cells, which expressed high E-cadherin levels; and “mesenchymal-like (M)” cells, which expressed low E-cadherin levels (Figure 4A). To identify genes within the SRAMs that distinguish E cells from M cells, we performed a Wilcoxon rank sum test. Out of the 578 unique genes, 111 (19%) were differentially methylated between E and M cells, with a *P*-value < 0.001. We called these genes EMT-SRAMs. A majority of EMT-SRAMs (N = 88) were methylated in M cells compared to E cells (Figure 4B, Additional file 6: Table S3). A few of the EMT-SRAMs, including *CDH1*, were already known to be epigenetically regulated during the process of EMT [19, 20]. However, the epigenetic regulation of the remaining EMT-SRAMs had not been established, including that of *SPINT1(HAI-1)* [21], *SYK* [22], *MST1R(RON)* [22] and *ESRP1* [23]. Interestingly, one of these genes is *AXL*, which encodes a receptor tyrosine kinase that is associated with EMT and with resistance to EGFR tyrosine kinase inhibition [24, 25]. We found that the expression of *AXL* was inversely correlated with promoter methylation (Figure 2), and its promoter methylation was positively correlated with E-cadherin expression (Additional file 7: Figure S4), indicating that *AXL* may be epigenetically regulated during EMT.

To further determine whether EMT-SRAMs define an epigenetically regulated group of genes relevant to EMT, we compared our gene list to a variety of publically available gene expression signatures from studies that experimentally manipulated cells to undergo EMT. Specifically, we examined datasets from prostate cancer cells transduced with *SNAI1* [26], a NSCLC epithelial cell line (H358) transduced with *SNAI1*, *ZEB1* or stimulated with TGF-beta [27], and HBME cells transduced with *Twist* [28]. We applied hypergeometric statistics to test the significance of overlap between the entire dataset of SRAMs (578 genes) and the EMT-SRAMs (111 genes). We found that the SRAMs significantly overlapped with the genes in the published datasets. On average, there was a 16% overlap between the SRAMs and published EMT gene signatures. EMT-SRAMs had greater overlap with the published EMT gene signatures, ranging from 18% in the breast cancer cell line signature to 48% in the H358 cells treated with TGF-beta (Additional file 8: Table S4). The majority of overlapping genes were downregulated, which is consistent with our observation of a greater proportion of genes being methylated in M cell lines. There appeared to be a greater overlap between genes from H358 cells and the SRAMs or the EMT-SRAMs than either the prostate or breast cancer datasets. This suggests that while there could be a common core group of genes for both the SRAMs and the EMT-associated SRAMs, the tissue-specific SRAMs may reflect the underlying biology of the tissue of origin.

Using a curated network extracted from Pathway Commons [29], which consists of 11,570 genes and over a million biological interactions, we performed a network analysis using markers of EMT, including 8 transcription factors and major regulators of EMT (*TWIST, SNAIL, SLUG, GSC, FOXC1, FOXC2, ZEB1* and *ZEB2*), and three recognized markers of EMT (*CDH1, CDH2* and *FN*), and the EMT-SRAM gene list. We were able to map 73/111 EMT-SRAMs. The pair-wise shortest distance between markers of EMT with EMT-SRAMs was calculated using Dijkastra’s algorithm [30]: 51/73 EMT-SRAMs (~70%) in the network had the shortest path lengths of 1 and 2, connecting at least one of the 11 markers of EMT (Additional file 9: Figure S5). This implies that a large proportion of the EMT-SRAMs are functionally related to regulators of EMT. Using hypergeometric statistics, we created a core EMT subnetwork (“EMT hub genes”) that accounted for all biological interactions between markers of EMT-linking genes (genes that are functionally related to EMT but are not core EMT factors or part of the EMT-SRAMs), and EMT-SRAMs (Figure 4C). While 2.8% of the genes in the entire curated network were cited in the literature as being related to EMT, 24.8% of the genes in the subnetwork were cited thusly (*P* < 0.0001, hypergeometric testing). This indicates that there was a significant enrichment for known markers of EMT in our subnetwork, as well as novel interactions.

Taken together, these findings demonstrate that a subset of NSCLC SRAMs is related to EMT, and that epigenetic regulation through DNA methylation may play an important role in the process of EMT.

### EMT-related genes are epigenetically regulated

**Figure 5:**
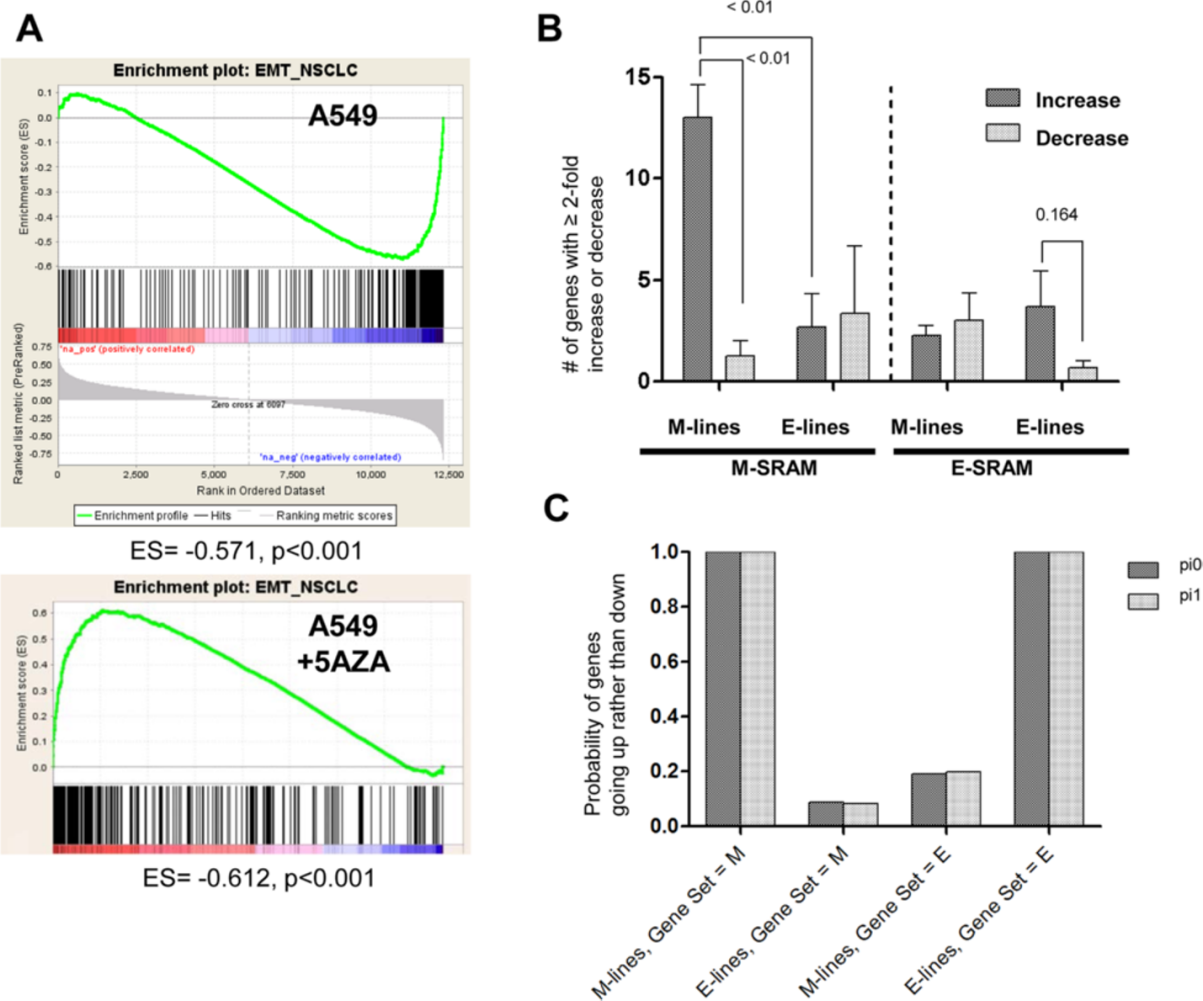
**SRAMs induced with 5AZA are strongly associated with M cell lines. A)** GSEA of SRAMs in A549 (M-type cell) finds a substantial proportion of genes to have a negative association with gene sets that are repressed during cell migration and EMT states; this relationship is readily reversed by 5AZA treatment. **B)** Data from Shames et al. [31] demonstrates more M-SRAMs being induced (≥ 2-fold more) than suppressed in M cells, which is not apparent in the E cells. A similar trend is noticed in the E cells for the E-SRAMs, although not significant. **C)** Bayesian statistical method to determine the posterior probability that more genes increase (rather than decrease) expression after treatment with 5AZA by the type of cell line, using either an uninformative prior (pi0) or a conservative prior (pi1).

To determine whether genes associated with EMT were epigenetically regulated, we created an EMT gene set based on the E-cadherin differential expression (high or low) in our panel of NSCLC cell lines. A total of 407 genes were ≥ 4-fold differentially expressed between these two groups and thus were included in the EMT gene set. We then identified the publicly available gene expression profiles of NSCLC cell lines before and after treatment with the hypomethylating agent 5-azacytidine (5AZA) from a single study [31]. We performed a GSEA using the A549 mesenchymal cell line. We found the EMT gene set to be negatively enriched when genes were ranked according to the rho value between gene expression and the average beta value in our integrative analysis (enrichment score -0.571, *P* < 0.001). We found the EMT gene set to be positively enriched when genes were ranked according to the log_2_ fold-change before and after treatment with 5AZA (enrichment score -0.612, *P* < 0.001) (Figure 5A). We found statistical significance (*P* < 0.001, hypergeometric testing) in the overlap between genes that were overexpressed in response to 5AZA and genes that were regulated by DNA promoter methylation and were represented in the EMT gene set enrichment.

Since the M cells have proportionally more SRAMs compared to E cells, we next determined whether 5AZA was upregulating a larger number of genes in M cells (A549, H157, H460, and H1299) compared to E cells (H1819, H1993, and H2347). We performed a GSEA that included the EMT gene set, with genes ranked according to the log_2_ fold-change before and after treatment with 5AZA, in all 7 NSCLC cell lines [31]. While the enrichment of the EMT gene set was significant for all but one E cell (H2347), the number of genes induced by 5AZA with a fold-change ≥ 2 and the enrichment scores tended to be higher in M cells (A549, H157, H460, H1299, Additional file 10: Figure S6A) compared to E cells (H1819, H1993, H2347, Additional file 10: Figure S6B) (Figure 5B). We confirmed these results with the dataset from Heller et al. [32] where two of the three cell lines (A549 and H1993) overlapped with that in the Shames et al. dataset. We performed GSEA analysis of these two lines and compared the 5AZA and 5AZA + trichostatin (TSA) treated cells compared to untreated cells. We found similar enrichment of the EMT gene set for the A549 cell line but not for the E cell type H1993 (Additional file 11: Table S5).

To further test the hypothesis that M-related SRAMs are more likely than E-related SRAMs to exhibit increased expression in M cells after 5AZA treatment, we used a Bayesian statistical method (Additional file 12: Text S1; Additional file 13: Table S6; Additional file 14: Table S7). We assigned each gene in each cell line to one of three categories (up, down, or constant), as defined by a change in expression that was at least a 2-fold change in response to 5AZA. We calculated the posterior probability that more genes increase (rather than decrease) expression after treatment with 5AZA, by the type of cell line, using either an uninformative prior or a conservative prior that assumes most genes do not change expression. We found that on average, the probability of induced gene expression in the M cells is high for the M-related SRAM category of genes, and is high for the E cells in the E-related SRAM category of genes (Figure 5C). These results corroborate the in silico data that indicated that more genes were methylated in the M cells and were inducible upon 5AZA treatment.

### Association between SRAM methylation and resistance to erlotinib in wild-type *EGFR/KRAS*NSCLC cell lines

**Figure 6:**
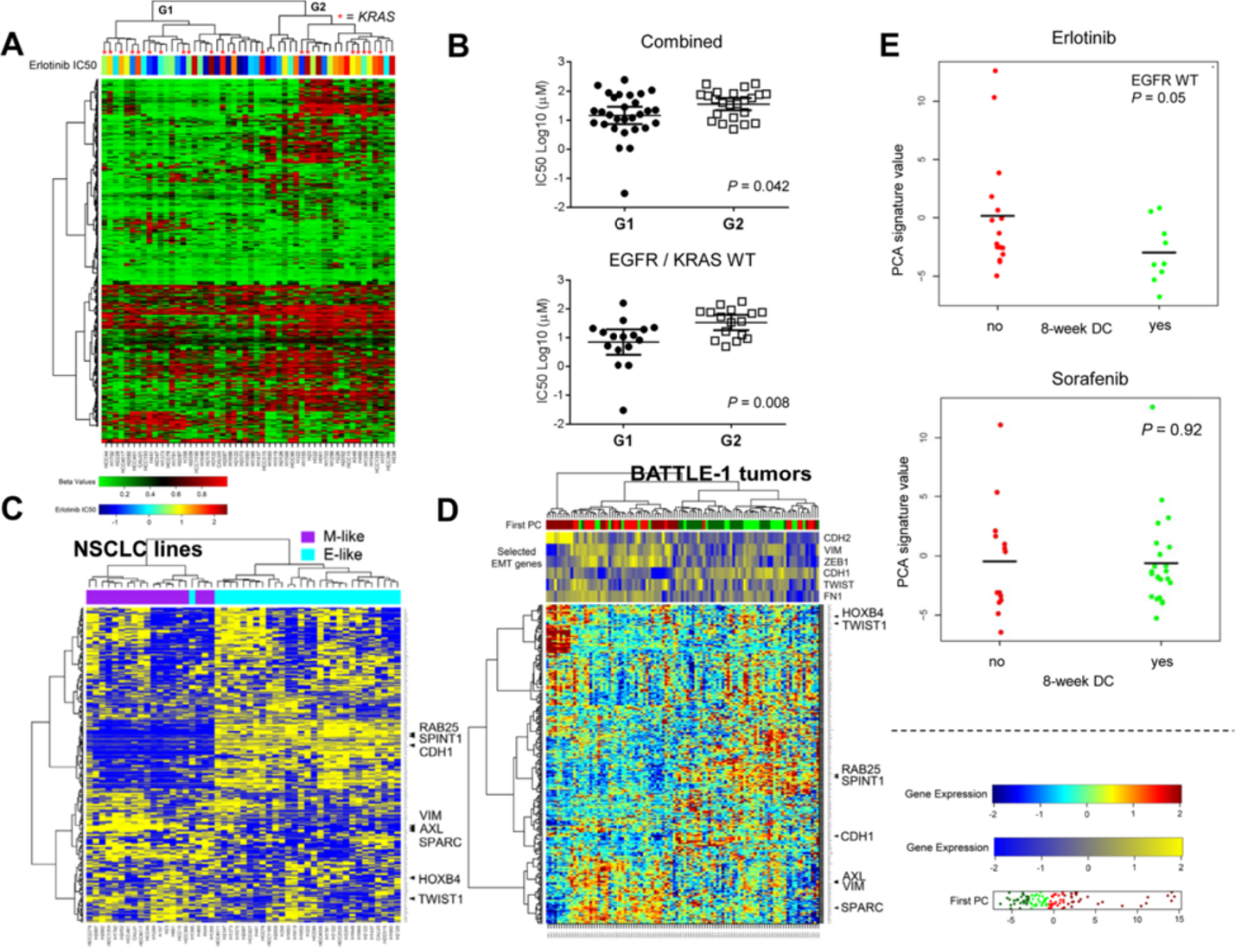
**Extent of methylated SRAMs associated with erlotinib response in cell lines and patients in BATTLE-1 trial. A)** Hierarchical clustering of EMT-SRAMs in 52 wild-type (WT) *EGFR* NSCLC cell lines, with erlotinib IC50 levels overlying individual cell lines. G1 and G2 represent the two cell lines clustered by SRAM methylation. *KRAS* mutant cells are represented by asterisks. **B)** Erlotinib IC50 values of G1 vs G2 clusters for the combined cell lines, and the WT *EGFR/KRAS* cells. G2 cluster had significantly higher IC50 values than G1 cluster. Error bars indicate average and 95% confidence interval. **C)** Hierarchical clustering of expression of SRAMs in the panel of cell lines with RPPA data for E-cadherin (49) clusters of E and M cells. Specific gene markers that relate to EMT are on the right. **D)** Hierarchical clustering based on first principle component analysis (PCA) of SRAM expression signature in the tumor biopsies from patients in BATTLE-1 trial clusters tumors into groups that resemble the separation seen in the cell lines. **E)** Eight-week disease control (DC) of patients treated with erlotinib or sorafenib based on the first PCA separation. *EGFR* mutant tumors were excluded.

Since the SRAMs segregate the cell lines into E and M cell types, and EMT has been implicated in erlotinib resistance [33, 34], we determined whether the two clusters of NSCLC cell lines defined by SRAMs (methylated and unmethylated EMT-SRAMs) could be associated with erlotinib resistance. Since *EGFR* mutation is known to be associated with sensitivity to erlotinib, and *KRAS* mutations are associated with resistance, we focused on the group of wild-type *EGFR/KRAS* cell lines. We found that the half maximal inhibitory concentration (IC_50_) for erlotinib was significantly higher in cell lines that segregated to clusters with methylated SRAMs compared to those that segregated to clusters with unmethylated SRAMs (Figures 6A and B).

### SRAM expression profiles in tumors segregate along E vs M expression patterns and erlotinib response

We next determined whether the SRAM profile was associated with clinical benefit in patients. Since we found no available clinical datasets with DNA methylation profiling and erlotinib drug response information, we analyzed the gene expression profile for SRAMs in baseline tumor biopsies collected in patients with stage IV NSCLC that were included in the clinical trial Biomarker-Integrated Approaches of Targeted Therapy for Lung Cancer Elimination (BATTLE-1) [35, 36]. Gene expression profiles were available for 27 patients treated with erlotinib (including 25 patients with wild-type *EGFR*), and for 47 patients treated with sorafenib (including 37 patients with wild-type *EGFR*), all of whom were evaulable for the primary endpoint of the trial, which was 8-week disease control. Interestingly, when SRAMs were used to cluster tumor samples collected at baseline in BATTLE-1, a similar pattern was observed *in vitro* (Figures 6C and D). In order to summarize the effect of the SRAMs, we computed the first principal component in each sample. Among the patients treated with erlotinib who had wild-type *EGFR*, the first principal component was lower in the patients who had a clinical status of disease control (*P* = 0.05) (Figure 6E). No difference was observed in patients treated with sorafenib (*P* = 0.92). When we further analyzed the 21 patients treated with erlotinib who had both *EGFR* and *KRAS* wild-type tumors, we found that the first principle component trended lower in patients with a clinical status of 8-week disease control (*P* = 0.08).

## Conclusions

In this study, we carried out an integrative analysis of gene expression and promoter CpG methylation profiles in NSCLC cell lines to identify a group of genes that were strongly associated with the degree of DNA methylation. We found that a subset of these epigenetically regulated genes reside within pathways related to epithelial and mesenchymal states of cancer. Many of the genes that distinguish E versus M cells in NSCLC significantly overlap with the genes that are suppressed during the EMT processes found in other cancer systems, such as prostate and breast cancers. We found that many of the genes were methylated in M cells, and that 5AZA upregulated genes that were silenced by DNA methylation in these cells compared to the epithelial counterpart. Furthermore, we found that SRAMs could predict erlotinib resistance in the NSCLC cell lines, and the gene expression signature of the SRAMs predicts erlotinib response in a cohort of patients with NSCLC who were treated with erlotinib. Our data suggest that the SRAMs of various tumors may hold biomarkers that are predictive of response to drug therapy.

Transcriptional regulation involves the interplay of a host of epigenetic factors, such as chromatin remodeling, histone modification, non-coding RNAs, and DNA methylation. Methylation of CpG sites promotes the recruitment of methyl-binding factors, which serves as a scaffold for protein complexes that contribute to nucleosome compaction, chromosomal condensation and transcriptional silencing [37]. While a promoter CpGi is classically linked to gene expression, the definition of what constitutes a CpGi is fairly arbitrary and based on computational prediction. Studies have shown that a large number of CG-rich areas within regions of the promoter and first exon that are not CpGi’s play important roles in transcriptional regulation [38]. We found that for probes corresponding to both CpG and non-CpG sites, a significant excess was seen with a rho cutoff of less than -0.5 than expected from normal distribution, suggesting the expression of genes with this type of inverse relationship with DNA methylation corresponds to the SRAMs. We also demonstrated from this data that there is an equal likelihood for SRAMs to correspond to promoters with CpGi’s as to those without CpGi’s. More than 80% of the CpG sites that are related to transcriptional regulation fall within 500 bp upstream or downstream of the TSS, which is consistent with what has been reported in the literature. Indeed, there were approximately 3.4% of the promoter CpG sites to correspond to SRAMs, and 578 genes with differential expression based on promoter CpG methylation in the NSCLC cell line to correspond to ~2.7% of the genome. This is somewhat similar to what has been reported in the literature regarding the percentage of genes induced after 5AZA treatment in multiple cancer types. However, for one study in NSCLC cell lines, the number of SRAMs we found appears to be somewhat lower [31]. This can be explained by the fact that 5AZA not only exerts gene induction through hypomethylation of normally hypermethylated promoters, but also has secondary effects, such as the DNA damage response [39]. Furthermore, our approach identified only genes that are differentially methylated and expressed between cell lines, and not genes that are uniformly suppressed in cancer cells by DNA methylation, such as *HIC1* [40] and *SOX17* [41], which may be induced by 5AZA. We attempted to identify genes whose differential methylation patterns could help cluster cells according to functional subgroups; therefore, we believe our approach identifies these differentially regulated genes. Indeed, as proof of the validity of our method, we identified many genes that are known to be differentially methylated across various cancer types (*CDKN2A*, *CDKN2B*, *MGMT*, *SFRP1*), in addition to many novel genes not previously known to be regulated by DNA methylation.

We found that nearly 20% of the lung cancer SRAMs are related to genes that distinguish epithelial from mesenchymal cell types, which is similar to what was recently reported [14, 42]. We compared our EMT-SRAM gene list with that from published gene expression datasets derived from various cell systems treated to undergo EMT, and found significant overlap in the gene sets. This was particularly apparent in the downregulated genes, suggesting that a significant proportion of genes that are downregulated during the EMT process may be regulated by DNA methylation. Using gene interaction network analysis, we found that almost 70% of the genes in the EMT-SRAM panel are one to two gene neighbors away from six of the key factors that play critical roles in the EMT process (*CDH1*, *FN1*, *CDH2*, *GSC*, *SNAI1*, *SNAI2*). A great majority of the methylated genes in the EMT-SRAM panel are seen in mesenchymal cells. This is consistent with data showing that the EMT state induces epigenetic alterations in numerous genes in a non-random fashion. In breast cancer, basal-like/mesenchymal breast cancers exhibit patterns of methylation in genes such as *Twist* and the estrogen receptor gene that are not found in the luminal type of cells [20]. E-cadherin, or *CDH1*, a gene involved in cell adhesion and signaling that plays a central role in EMT, is suppressed by DNA methylation during permanent and irreversible EMT. This is seen for Ras-transformed breast cancer cells stimulated with serum. EMT induced in breast cancer cells by treatment with isolated TGF-beta was found to be reversible when the transforming growth factor was removed [20]. However, when non-transformed AML12 hepatocytes were induced to undergo EMT with TGF-beta, DNA methylation was not observed, even though abundant epigenetic reprogramming is known to occur in specific chromatin regions throughout the genome [43]. These data support the proposition that DNA methylation of EMT-related factors is a non-random event that depends on the transient versus permanent state of EMT. While similar sets of genes may be suppressed during transient EMT, this is not due to DNA methylation but rather is regulated by chromatin factors. However, when subject to certain long-term environmental conditions as well as oncogenic factors, these EMT-related genes become progressively and irreversibly methylated [14]. Cells that acquire these characteristics are locked into an epithelial-like or mesenchymal-like state, which is seen in established cell lines of various origins and also in primary tumors. Tumors that are locked into mesenchymal-like states may be responsible for intrinsic resistance to drug therapies, such as erlotinib. The mesenchymal-associated SRAMs could unveil important pathways involved in the adaptive regulation of genes directly involved in drug sensitivity, such as the expression of *AXL* in erlotinib resistance.

We found that 5AZA treatment induced many of the genes that were silenced by DNA methylation, and that there was a preferential induction of genes in the mesenchymal cells, although a great majority of the genes were uninducible, as seen in breast cancer [14]. Erlotinib therapy in combination with epigenetic agents may be a promising avenue to help reverse resistance to EGFR tyrosine kinase inhibitors in wild-type *EGFR* cells, as shown in a study of cell lines, where EGFR silencing by DNA methylation contributed to gefitinib resistance that was reversed with decitabine treatment [44]. While current uses of decitabine are mainly reserved for hematologic malignancies, the future use of this drug in solid tumors may require a combination of epigenetic agents, such as histone deacetylase inhibitors with decitabine, or selective small molecular DNA methyltransferase inhibitors.

## Methods

### Cell lines and tumors

Additional file 15: Table S8 lists the 73 NSCLC and normal cell lines used in this study. Cells were grown to logarithmic phase and collected at 70–80% confluence in growth media supplemented with 10% fetal bovine serum. Total RNA was extracted using Trizol reagent. DNA was collected using QIAquick DNA extraction kit (Qiagen, Valencia, CA). Protein lysates used for RPPA were collected using RPPA lysis buffer and protein quantitation using BCA.

Tumor samples were acquired from surgical excision of the tumor mass along with normal lung within the lobectomy specimen. Small tissue blocks were snap-frozen and stored at -80°C. The cohort included only patients from Kyoto University hospital, with the following patient and tumor characteristics: Median age = 67.5 years, males = 61.4%, never smokers = 32.5%, adenocarcinomas = 69.3%, squamous cell carcinoma = 24.6%, large cell = 2.6%, or NOS = 3.5%, Well/Mod/Poorly differentiated tumors = 31.7%/50.0%/18.4%, stage I-II = 75.5%, stage III = 20.2%, or stage IV = 3.5%, and EGFR mutants = 35.1%.

### Genome-wide DNA methylation analysis at promoter CpG sites

DNA methylation status of a set of 27,579 CpG sites around promoters of 14,475 consensus coding sequences was interrogated using the Illumina HumanMethylation27 BeadChip (Illumina Inc, San Diego, CA). Genomic DNA (1 μg) extracted from NSCLC cell lines or tumors was bisulfite converted using EZ DNA Methylation kit (Zymo Research Corp, Orange, CA), and utilizing a cyclic denaturation step during the conversion reaction as suggested by Illumina for optimal conversion efficiency. Whole-genome amplification, fragmentation, hybridization, washing, counterstaining, and scanning were performed according to the manufacturer’s instructions. The scanner data and image output files were managed with the Illumina BeadStudio Methylation Module v.3.2. The normalized data, presented as beta values, represent the degree of methylation at each CpG site, with 0 being unmethylated, and 1 being methylated.

### MRNA gene expression data

Illumina HumanWG-6 v2 BeadChip human whole-genome expression arrays (Illumina, Inc., San Diego, CA) were used for mRNA expression profiling. The platform contains 48,700 probes. Each RNA sample was amplified using the Ambion TotalPrep RNA amplification kit with biotin UTP (Enzo) labeling (Applied Biosystems/Ambion, Austin, TX). The Ambion Illumina RNA amplification kit uses the T7 oligo(dT) primer to generate single-stranded cDNA followed by a second strand synthesis to generate double-stranded cDNA, which is then column purified. *In vitro* transcription was performed to synthesize biotin-labeled cRNA using T7 RNA polymerase, and the cRNA was column purified. The cRNA was then checked for size and yield using the Bio-Rad Experion system (Bio-Rad Laboratories, Hercules, CA). For each array, 1.5 μg of cRNA was hybridized by using standard Illumina protocols, with streptavidin-Cy3 for detection. Slides were scanned using an Illumina BeadStation scanner. Expression values were extracted using Illumina BeadStudio v2. The data were background subtracted using the model-based background correction for BeadArrays algorithm [45, 46] and were quantile-normalized.

### Bioinformatics preliminaries

A crucial step for integrative analysis of genomic data across platforms is to include only genes that appear on both platforms and to update the gene annotation. We fitted the probes without the associated gene symbols. For the two platforms, we assumed that the GenBank accession numbers supplied by the array manufacturer were accurate. Using these accession numbers, we updated the annotation for both platforms to the recent UniGene build, using SOURCE, provided by Stanford University [47].

### Correlating DNA methylation with mRNA expression measurements on matched genes

On the Illumina mRNA expression platform, for a gene with multiple measurements (multiple probes), we computed the Pearson correlation coefficients and then averaged the measurements with sufficient correlation coefficients (R ≥ 0.4). Once this was completed, we ended up with 16,104 unique genes on the mRNA platform. For the DNA methylation platform, since each probe corresponded to a unique CpG site at the promoter region, we preserved all the measurements irrespective of the number of probes per gene symbol. We matched 22,992 CpG methylation measurements, representing 12,313 unique genes. We then computed the rank correlation and associated *P* values for each gene between the degree of methylation and the associated gene expression. We modeled the resulting *P* values using a beta-uniform mixture model. We selected the appropriate false discovery rate cutoff to identify the set of genes significantly repressed in association with methylation (SRAMs) based on the genes that had statistically significant correlation in the degree of methylation and the associated mRNA expression level.

### Pyrosequencing

Bisulfite treatment of genomic DNA was performed using the EpiTect bisulfite kit (Qiagen, Valencia, CA) according to the manufacturer’s instructions. Pyrosequencing was carried out for 8 of the SRAMs, reflecting both promoter or first exon CpGi’s (*CBLC, MST1R, LAD1, ESRP1, HOXB4*) and non-CpGi genes (*PRSS8, RAB25, AXL*) (Additional file 16: Table S9). One microliter of bisulfite-treated DNA was used for each polymerase chain reaction (PCR). After an initial hot-start at 95°C for 5 minutes, all PCR reactions ran at 95°C for 30 seconds, annealing at various temperatures for 30 seconds, and underwent an extension step at 72°C for 30 seconds. All reactions were carried out with a nested PCR step during which a biotinylated universal primer was added. After PCR, the biotinylated strand was captured on streptavidin-coated beads (Amersham Bioscience, Uppsala, Sweden) and incubated with sequencing primers. Pyrosequencing was performed with PSQ HS 96 Gold reagents on a PS QHS 96 pyrosequencer (Biotage, Uppsala, Sweden) as published previously [48].

### Quantitative real-time PCR (qPCR)

RNA was isolated from cell culture cells growing in logarithmic phase (70%) using Trizol Reagent (Invitrogen, Carlsbad, CA). First-strand cDNA synthesis was performed using 1 microgram of RNA with the SuperScript III First Strand synthesis kit for RT-PCR (Invitrogen, Carlsbad, CA). Triplicates of qPCR were performed using the QuantiTect SYBR Green PCR kit (Qiagen, Valencia, CA; primer sequence in Additional file 15: Table S8) on the ABI7500 Fast Real-Time PCR system. Normalization was carried out using *GAPDH* as a reference gene.

### Gene set enrichment analysis (GSEA)

Functional analyses were performed using GSEA software v3.7 [17]. GSEA is a robust computational method that determines whether an a priori defined set of genes shows statistically significant, concordant differences between two biologic states. GSEA eases the interpretation of large-scale expression data by identifying pathways and processes. This method shifts the level of analysis of the microarray experiment from single genes to sets of related genes. The main advantage of this method is its flexibility in creating a molecular signature database of gene sets. Such a database of gene sets allows biologist to make use of previously accumulated biologic knowledge in the analysis and makes a more biology-driven analysis of microarray data possible. For the GSEA, the following three required data inputs were generated: (1) genes pre-ranked according to the Pearson correlation between DNA methylation and gene expression, (2) a mapping file for the identification of the Illumina HumanMethylation27 BeadChip platform, and (3) the C2 catalog of curated gene sets from the Molecular Signature Database [49]. In the analysis, we included chemical and genetic perturbations, canonical pathways, Biocarta gene sets, KEGG gene sets, and Reactome gene sets. After collapsing the probesets into gene symbols, 12,313 genes were considered. Default parameters were used throughout (we set the inclusion gene size between 15 and 500, and permutated the phenotype 1,000 times). A total of 2,319 (or 2,320 if we include the EMT gene set) gene sets were included in the analysis.

### EMT network analysis

Known EMT genes (n = 11) were mapped along with the genes identified in our EMT-SRAM signature in a curated network downloaded from Pathway Commons [29], which consists of 11,570 genes and over a million biological interactions. The pair-wise shortest distances of the 11 known EMT genes with our EMT signature genes in the network were calculated using Dijkstra’s algorithm [30]. We then applied the following strategy to create a core, enriched subnetwork that consisted of biological interactions between EMT regulatory factors and our EMT signature genes. We first applied hypergeometric testing to identify linking genes that were not in the EMT signature list, but which are statistically enriched for connections to members of the signature gene list with the 11 EMT factor genes. Linking genes that pass a *P*-value threshold of 0.05 were included in the analysis as significantly connected with the EMT signature genes and the known EMT-related factors. All interactions among the signature genes, linking genes, and the EMT factor genes were included to create the EMT subnetwork. The finalized core network was visualized using Cytoscape [50].

To determine the relevance of the genes included in the subnetwork analysis, we used the genes cited in the EMT literature to test whether our subnetwork enriched for EMT-related genes. We extracted all Pubmed IDs related to the term “Epithelial Mesenchymal Transition” from Pubmed. For each gene, we calculated the number of citations related to EMT by mapping the extracted Pubmed IDs to the gene citation information from Entrez Gene, composed of genes and their corresponding cited literature [51]. We then applied hypergeometric testing to determine whether there was a significant enrichment of EMT-related genes in our subnetwork.

### Availability of supporting data

All human cell lines used in this study were produced by Dr. John Minna and are all publically available through ATCC. The data sets supporting the results of this article are included within the article and in the supplementary tables.

### Ethics statement

Written informed consents were obtained from patients by approval of Kyoto University Ethics Committee for the use of the clinical samples under protocol G358 entitled “Epigenomic analysis of the genes specific for nodular or distant metastatic abilities in lung cancer clinical samples.” The University of Texas MD Anderson Cancer Center IRB has waived the need for consent for the use of the clinical samples reported in this study because all samples were acquired from Kyoto University and fully de-identified to meet HIPAA regulations. The MD Anderson IRB has given approval for the use of all clinical samples on protocol LAB09-0841. All human cell lines used in this study were produced by Dr. John Minna and are all publically available through ATCC.

## Electronic supplementary material

Additional file 1: Figure S1: Integrative analysis of gene expression and methylation degree for *CDKN2A.* A) Four probes interrogating 4 CpG sites around the promoter CpG island region, mapped using the UCSC Genome Browser. B) All 4 probes correspond to CpG sites that have strongly negative correlation with gene expression, with rho values < -0.5. (TIFF 547 KB)

Additional file 2: Table S1: List of 750 probes corresponding to 578 unique NSCLC SRAMs using FDR cutoff of 0.005. (XLSX 65 KB)

Additional file 3: Figure S2: Representative candidate SRAMs. Some SRAMs with rho ≤ -0.5 out of a set of 750 probes that correspond to 578 unique genes. The x-axis is the degree of methylation, expressed as a beta value from 0 to 1, with 1 indicating full methylation. The y-axis is the gene expression level from the Illumina HumanWG-6 v 2 BeadChip, expressed on a log2 scale. (TIFF 964 KB)

Additional file 4: Figure S3: Experimental validation of a subset of SRAMs. Using pyrosequencing of bisulfite-treated DNA and real-time PCR, we aimed to validate the results obtained in the array analysis. The expression of these genes is not known to be regulated by DNA methylation. A) Correlation plots from our integrative analysis of 5 genes. B) Correlation between the Infinium methylation array beta value and pyrosequencing methylation level at each promoter region. C) Relationship between gene expression levels using real-time PCR and the degree of methylation determined by pyrosequencing. Note that the findings are consistent with the array data. (TIFF 715 KB)

Additional file 5: Table S2: Top 7 ranked gene lists in gene set enrichment analysis of the SRAM list of genes. (XLSX 15 KB)

Additional file 6: Table S3: List of 135 probes corresponding to 111 unique SRAMs that discriminate E and E cells. (XLSX 18 KB)

Additional file 7: Figure S4: Heatmap of the methylation status of a few EMT-SRAMs. Three genes represent the genes that are preferentially methylated (and silenced) in E-cadherin-low cells (*SPINT1, RAB25, CDH1*); three genes are preferentially methylated in E-cadherin-high cells (*AXL, TWIST1, SPARC*). The number in the parentheses is the number of CpG probes that represent the data for each gene. (TIFF 1 MB)

Additional file 8: Table S4: Overlap between EMT and breast cancer datasets with lists of SRAMs or EMT-SRAMs. (DOCX 16 KB)

Additional file 9: Figure S5: EMT-SRAM association with specific hub genes using curated network analysis. A – E) Closest second neighbor network representation with each of the EMT factors (hub genes), demonstrating that *CDH1* acts as a central hub for a majority of the EMT-related factors, with strong connections with *FN1* and *CDH2. SNAI1*/*2*/*GSC* genes have secondary networks that are not as closely tied to the other three factors. (TIFF 2 MB)

Additional file 10: Figure S6: EMT-NSCLC gene set enrichment in NSCLC cell lines from the Shames *et al.* dataset [31] treated with 1000 μM 5AZA. A) Gene set enrichment analysis (GSEA) of 5AZA-treated mesenchymal cell lines (*n* = 4) enriches for the EMT-NSCLC gene set in a positive direction, with low *P* values and false discovery rate (FDR) q-values. B) GSEA of 5AZA-treated epithelial cell lines (*n* = 3) also enriches for genes present in the EMT-NSCLC gene set, but with much higher FDR q-values. (TIFF 1 MB)

Additional file 11: Table S5: GSEA using EMT gene set for two cell lines from Heller et al. [32]. (DOCX 15 KB)

Additional file 12: Text S1: Supporting Information. (DOCX 18 KB)

Additional file 13: Table S6: Posterior probability that more genes increase (rather than decrease) expression after treatment with 5AZA, by type of cell line, using either an uninformative prior or a conservative prior that assumes most genes do not change expression. (DOCX 18 KB)

Additional file 14: Table S7: Posterior probability that more genes increase (rather than decrease) expression in a cell line after treatment with 5AZA, using either an uninformative prior or a conservative prior that assumes most genes do not change expression. (DOCX 18 KB)

Additional file 15: Table S8: Characteristics of the 73 cell lines used in the study: the ATCC alias, histologic origin, and EGFR mutation status. (XLSX 12 KB)

Additional file 16: Table S9: Primer sets used for validation of gene expression by quantitative RT-PCR (QPCR) and pyrosequencing (PSQ). (XLSX 30 KB)

## Abbreviations

5AZA: 5-azacytidine
BATTLE-1 trial: Biomarker-Integrated Approaches of Targeted Therapy for Lung Cancer Elimination trial
CpG: Cytosine bonded to guanine with a phosphodiester bond
CpGi: CpG islands
E cells: Epithelial-like cells
EMT: Epithelial-to-mesenchymal transition
GSEA: Gene set enrichment analysis
M cells: Mesenchymal-like cells
MGMT: O6-methylguanine-DNA methyltransferase
NSCLC: Non-small cell lung cancer
PCR: Polymerase chain reaction
qPCR: Quantitative real-time PCR
SRAM: Gene for which expression is significantly repressed in association with DNA methylation
TSS: Transcription starting site.
