# Supplementary figures and images for: Genes suppressed by DNA methylation in non-small cell lung cancer reveal the epigenetics of epithelial–mesenchymal transition
Source: BMC Genomics. 2014 Dec 8;15(1):1079. doi: 10.1186/1471-2164-15-1079 (PMC4298954; doi:10.1186/1471-2164-15-1079)

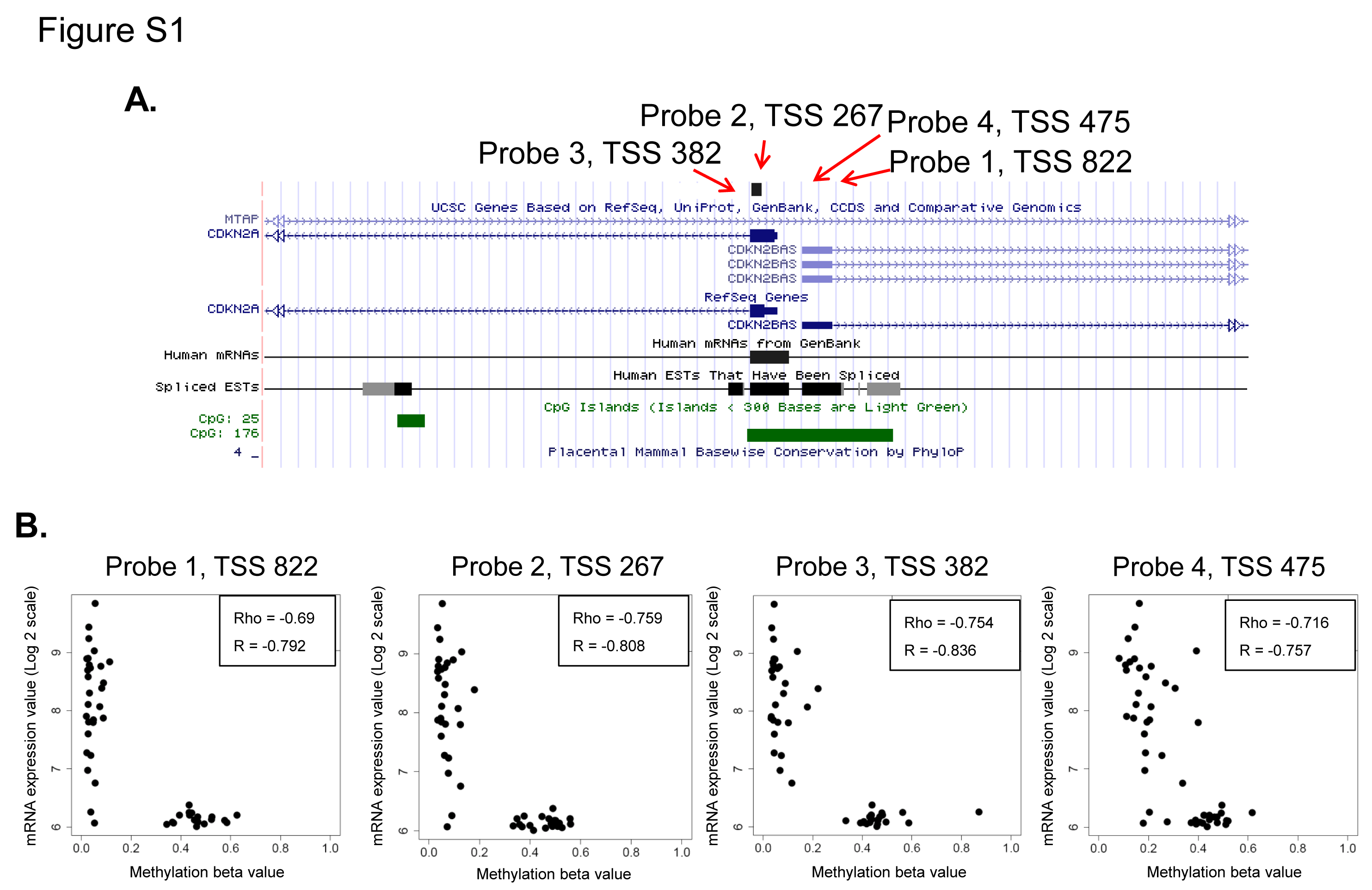

Supplement: Supplementary file 1 — Additional file 1: Figure S1: Integrative analysis of gene expression and methylation degree for CDKN2A. A) Four probes interrogating 4 CpG sites around the promoter CpG island region, mapped using the UCSC Genome Browser. B) All 4 probes correspond to CpG sites that have strongly negative correlation with gene expression, with rho values < -0.5. (TIFF 547 KB) [file 12864_2014_6772_MOESM1_ESM.tiff]

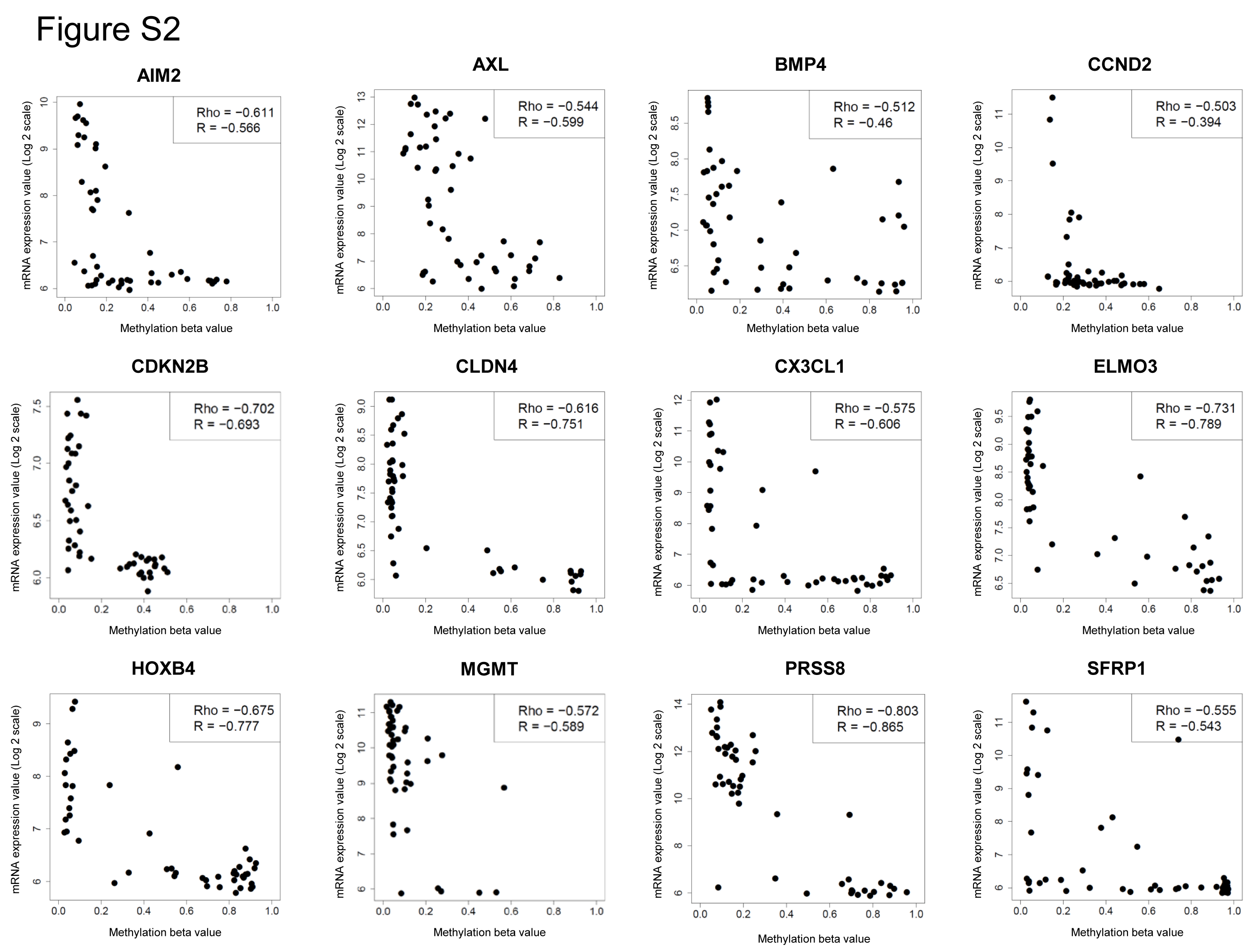

Supplement: Supplementary file 3 — Additional file 3: Figure S2: Representative candidate SRAMs. Some SRAMs with rho ≤ -0.5 out of a set of 750 probes that correspond to 578 unique genes. The x-axis is the degree of methylation, expressed as a beta value from 0 to 1, with 1 indicating full methylation. The y-axis is the gene expression level from the Illumina HumanWG-6 v 2 BeadChip, expressed on a log2 scale. (TIFF 964 KB) [file 12864_2014_6772_MOESM3_ESM.tiff]

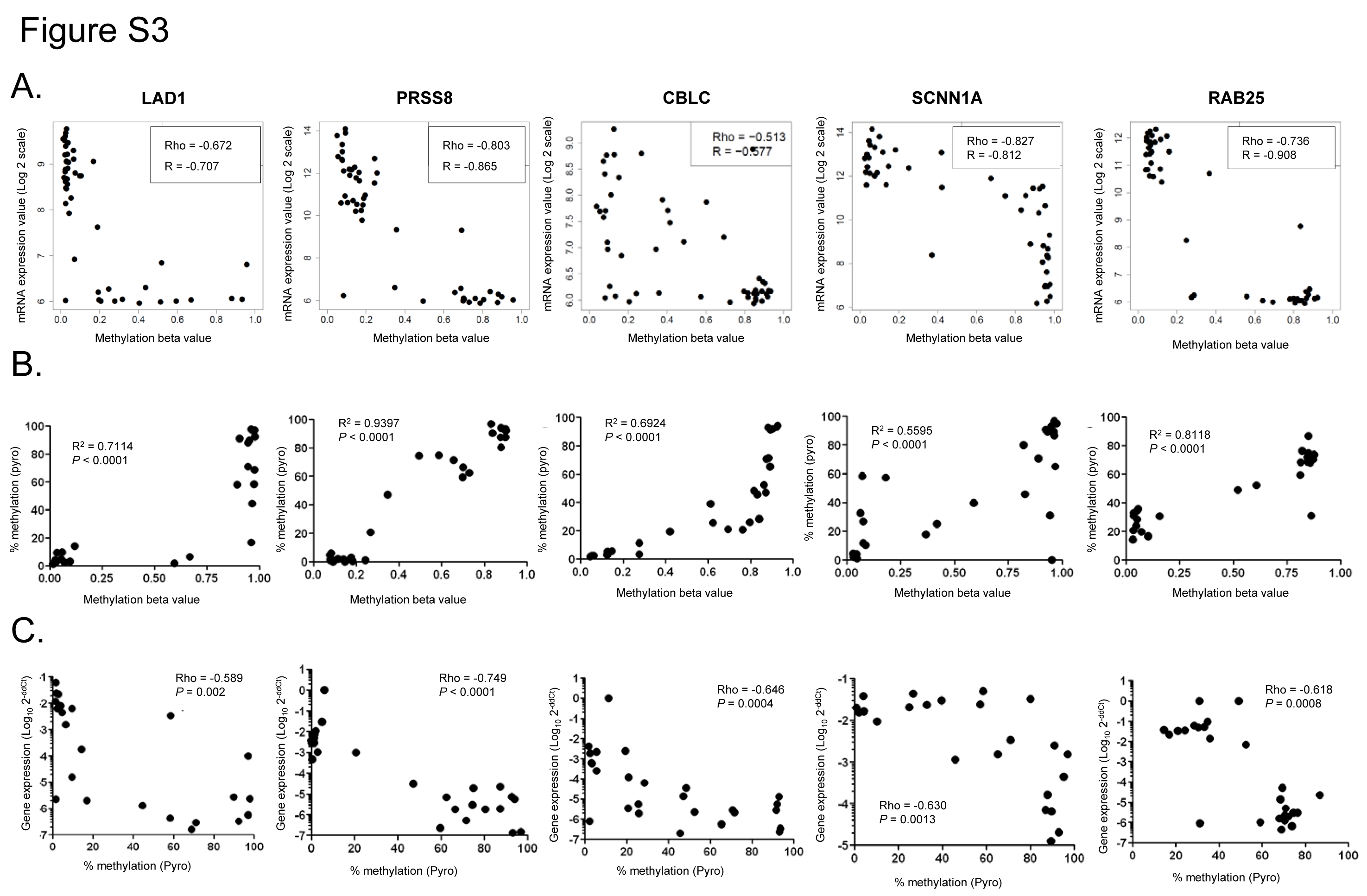

Supplement: Supplementary file 4 — Additional file 4: Figure S3: Experimental validation of a subset of SRAMs. Using pyrosequencing of bisulfite-treated DNA and real-time PCR, we aimed to validate the results obtained in the array analysis. The expression of these genes is not known to be regulated by DNA methylation. A) Correlation plots from our integrative analysis of 5 genes. B) Correlation between the Infinium methylation array beta value and pyrosequencing methylation level at each promoter region. C) Relationship between gene expression levels using real-time PCR and the degree of methylation determined by pyrosequencing. Note that the findings are consistent with the array data. (TIFF 715 KB) [file 12864_2014_6772_MOESM4_ESM.tiff]

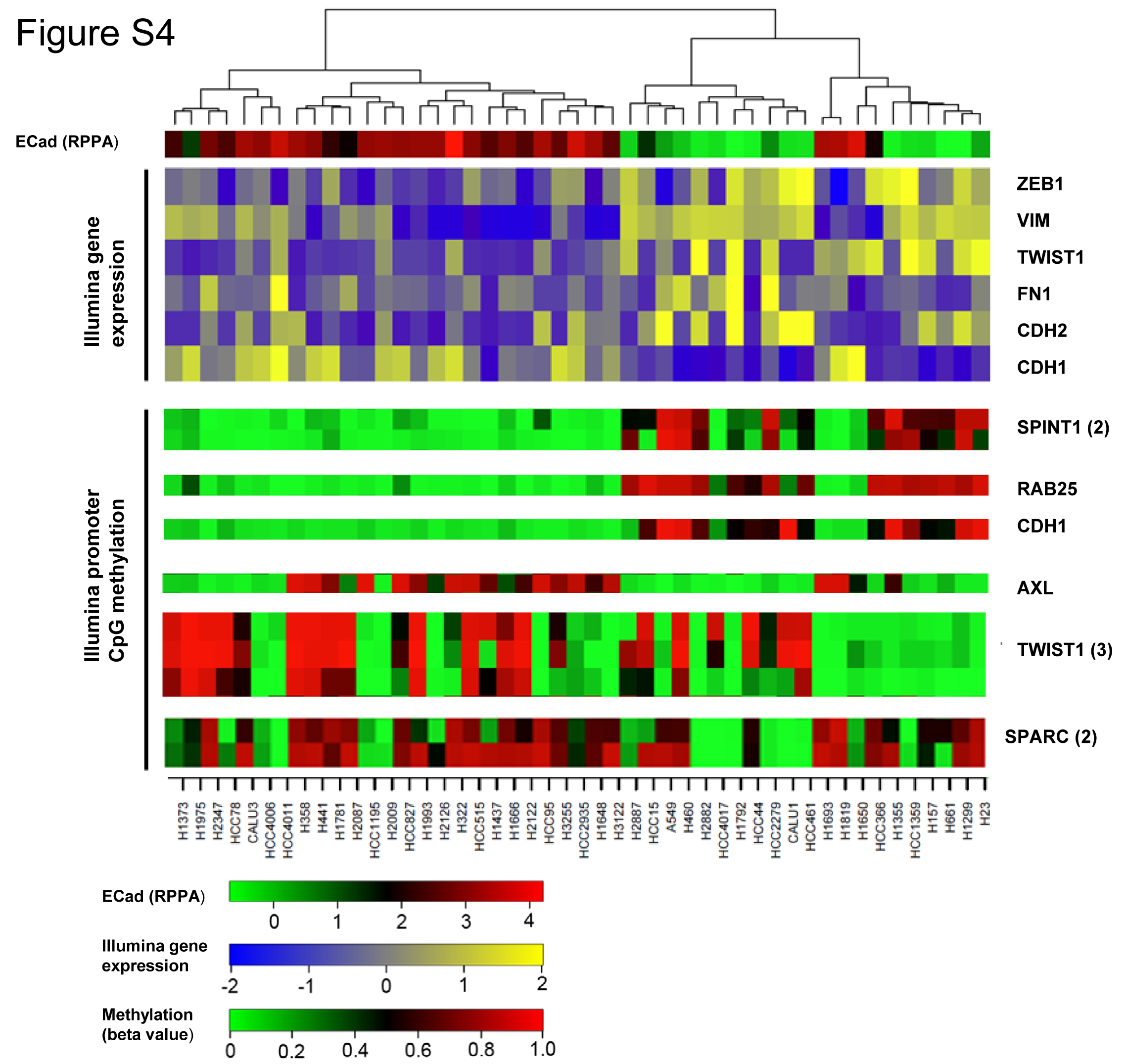

Supplement: Supplementary file 7 — Additional file 7: Figure S4: Heatmap of the methylation status of a few EMT-SRAMs. Three genes represent the genes that are preferentially methylated (and silenced) in E-cadherin-low cells (SPINT1, RAB25, CDH1); three genes are preferentially methylated in E-cadherin-high cells (AXL, TWIST1, SPARC). The number in the parentheses is the number of CpG probes that represent the data for each gene. (TIFF 1 MB) [file 12864_2014_6772_MOESM7_ESM.tiff]

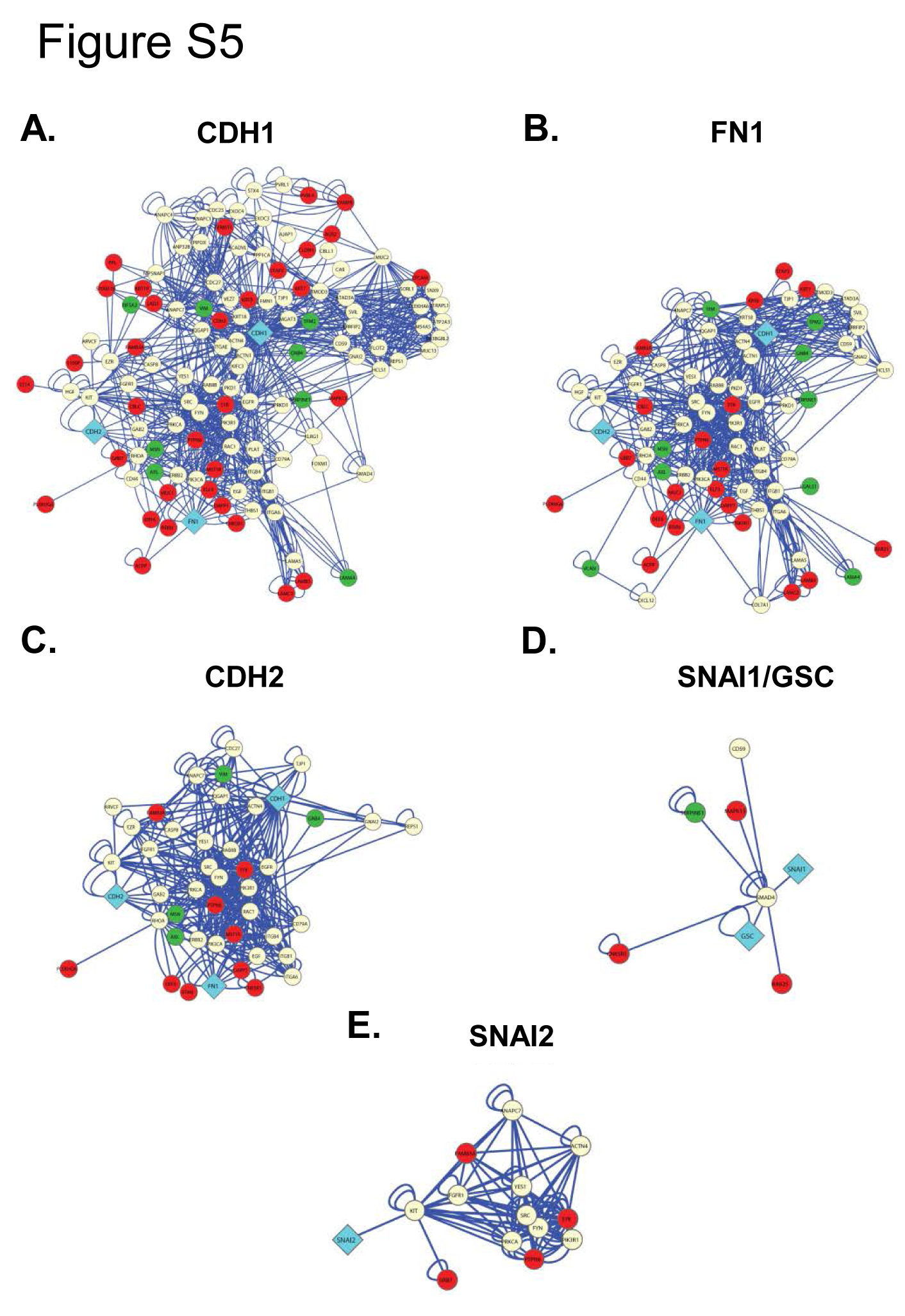

Supplement: Supplementary file 9 — Additional file 9: Figure S5: EMT-SRAM association with specific hub genes using curated network analysis. A – E) Closest second neighbor network representation with each of the EMT factors (hub genes), demonstrating that CDH1 acts as a central hub for a majority of the EMT-related factors, with strong connections with FN1 and CDH2. SNAI1/2/GSC genes have secondary networks that are not as closely tied to the other three factors. (TIFF 2 MB) [file 12864_2014_6772_MOESM9_ESM.tiff]

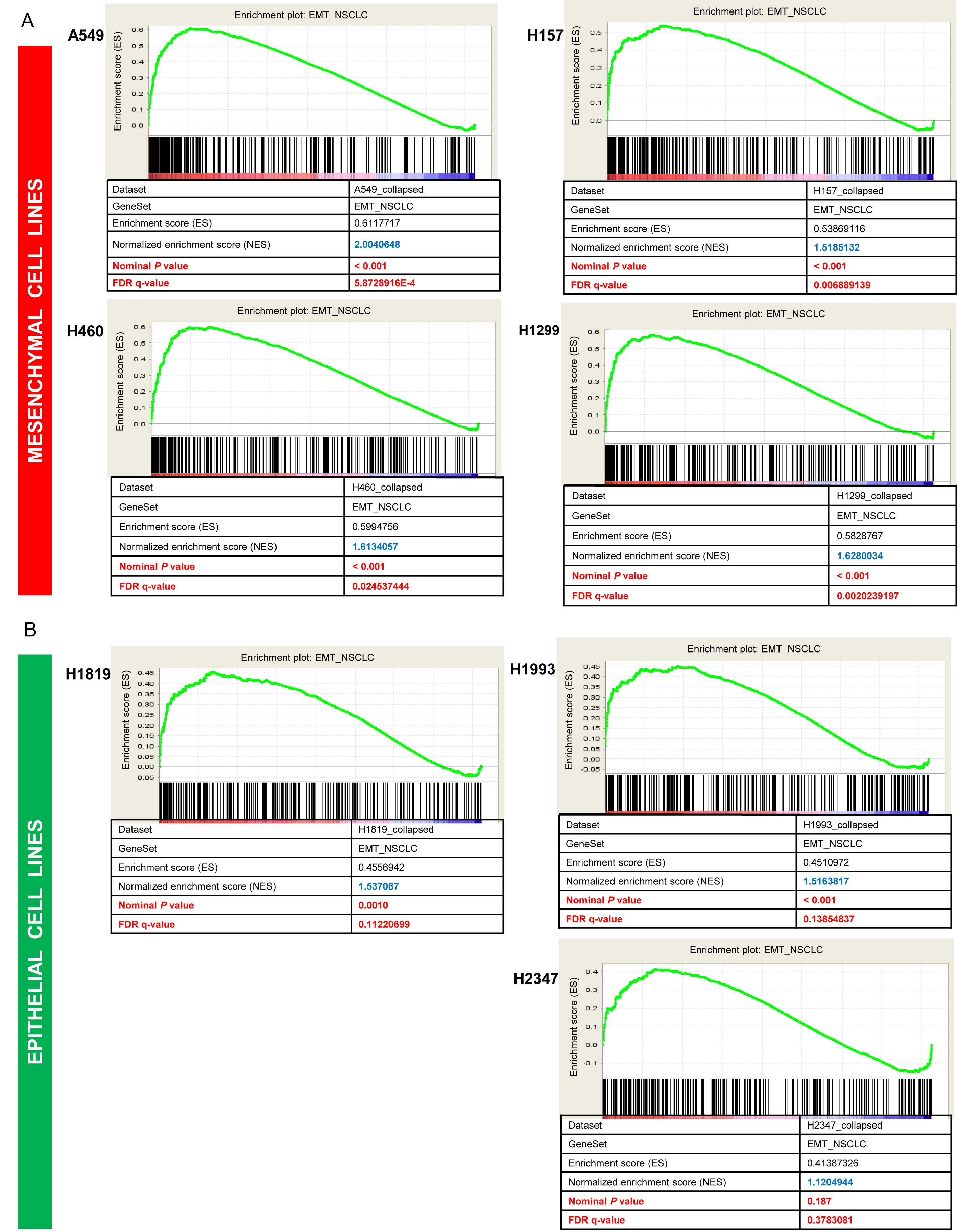

Supplement: Supplementary file 10 — Additional file 10: Figure S6: EMT-NSCLC gene set enrichment in NSCLC cell lines from the Shames et al. dataset [31] treated with 1000 μM 5AZA. A) Gene set enrichment analysis (GSEA) of 5AZA-treated mesenchymal cell lines (n = 4) enriches for the EMT-NSCLC gene set in a positive direction, with low P values and false discovery rate (FDR) q-values. B) GSEA of 5AZA-treated epithelial cell lines (n = 3) also enriches for genes present in the EMT-NSCLC gene set, but with much higher FDR q-values. (TIFF 1 MB) [file 12864_2014_6772_MOESM10_ESM.tiff]
