# Supplementary material for: Genes suppressed by DNA methylation in non-small cell lung cancer reveal the epigenetics of epithelial–mesenchymal transition
Source: BMC Genomics. 2014 Dec 8;15(1):1079. doi: 10.1186/1471-2164-15-1079 (PMC4298954; doi:10.1186/1471-2164-15-1079)
Supplement: Supplementary file 8 — Additional file 8: Table S4: Overlap between EMT and breast cancer datasets with lists of SRAMs or EMT-SRAMs. (DOCX 16 KB) [file 12864_2014_6772_MOESM8_ESM.docx]

**Table S4. Overlap between EMT and breast cancer datasets with lists of SRAMs or EMT-SRAMs.**

| **Category** | **Prostate EMT** | **H358 SNAIL** | **H358 TGFbeta** | **H358 ZEB1** | **Breast EMT** | **Luminal vs Basal** | **Luminal vs Mesenchymal** | **Basal vs Mesenchymal** | **Breast Cancer Methylation** |
| --- | --- | --- | --- | --- | --- | --- | --- | --- | --- |
| SRAM Overlap size | 82 | 90 | 110 | 103 | 46 | 58 | 93 | 32 | 52 |
| Overlap % | 14.19% | 15.57% | 19.03% | 17.82% | 7.96% | 10.03% | 16.09% | 5.54% | 9.00% |
| SRAM Overlap |  |  |  |  | 9.99 | 4.75 |  |  |  |
| *P* value | 0 | 0 | 0 | 0 | E-015 | E-08 | 0 | 0 | 0 |
|  |  |  |  |  |  |  |  |  |  |
| EMT-SRAM overlap size | 45 | 37 | 53 | 50 | 20 | 12 | 40 | 26 | 19 |
| EMT-SRAM Overlap % | 40.54% | 33.33% | 47.75% | 45.05% | 18.02% | 10.81% | 36.04% | 23.42% | 17.12% |
| EMT-SRAM |  |  |  |  | 7.41 |  |  |  |  |
| Overlap *P* value | 0 | 0 | 0 | 0 | E-014 | 0.0015 | 0 | 0 | 0 |

EMT = epithelial–mesenchymal transition; SRAMs = genes significantly repressed in association with DNA methylation; EMT-SRAMs = genes differentially methylated between epithelial and mesenchymal cells

*P* value testing by hypergeometric analysis. Overlap % is calculated by the ratio of the overlap size compared to the size of the SRAM and EMT-SRAM.
