# Supplementary material for: Genes suppressed by DNA methylation in non-small cell lung cancer reveal the epigenetics of epithelial–mesenchymal transition
Source: BMC Genomics. 2014 Dec 8;15(1):1079. doi: 10.1186/1471-2164-15-1079 (PMC4298954; doi:10.1186/1471-2164-15-1079)
Supplement: Supplementary file 11 — Additional file 11: Table S5: GSEA using EMT gene set for two cell lines from Heller et al. [32]. (DOCX 15 KB) [file 12864_2014_6772_MOESM11_ESM.docx]

| **Cell line and condition*** | **EMT status** | **NAME** | **SIZE** | **ES** | **NES** | **NOM p-val** | **FDR q-val** | **EMT geneset rank out of 3421 genesets** |
| --- | --- | --- | --- | --- | --- | --- | --- | --- |
| A549_A_vs_U | Mesenchymal | EMT_NSCLC | 71 | 0.5259 | 1.5087 | 0.0073 | 0.0662 | 727 |
| A549_AT_vs_U | mesenchymal | EMT_NSCLC | 71 | 0.5121 | 1.4783 | 0.0095 | 0.0745 | 826 |
| H1993_A_vs_U | Epithelial | EMT_NSCLC | 71 | 0.3132 | 1.0480 | 0.4209 | 0.5736 | 2497 |
| H1993_AT_vs_U | Epithelial | EMT_NSCLC | 71 | 0.4822 | 1.3834 | 0.0382 | 0.1264 | 1179 |
|  |  |  |  |  |  |  |  |  |
| *A = 5AZA; U=Untreated; AT=5AZA+TSA | | |  |  |  |  |  |  |

**Table S5. GSEA using EMT gene set for two cell lines from Heller et al. [32]**
