# Supplementary material for: Genes suppressed by DNA methylation in non-small cell lung cancer reveal the epigenetics of epithelial–mesenchymal transition
Source: BMC Genomics. 2014 Dec 8;15(1):1079. doi: 10.1186/1471-2164-15-1079 (PMC4298954; doi:10.1186/1471-2164-15-1079)
Supplement: Supplementary file 12 — Additional file 12: Text S1: Supporting Information. (DOCX 18 KB) [file 12864_2014_6772_MOESM12_ESM.docx]

**Genes suppressed by DNA methylation in non-small cell lung cancer reveal the epigenetics of epithelial-mesenchymal transition**

**Text S1**

**Supporting Information**

**SI Methods**

Note: SRAMs = Genes significantly repressed in association with DNA methylation

We used a Bayesian statistical method to test the hypothesis that mesenchymal-SRAMs are more likely than epithelial-SRAMs to exhibit increased gene expression in mesenchymal-type cell lines after treatment with 5AZA. Each gene in each cell line was assigned to one of three categories (U=up, D=down, or C=constant), defined by a change in gene expression of at least 2-fold in response to treatment with 5AZA. The triplet n = (n_U_, n_D_, n_C_) consisting of counts of the genes in the three categories was modeled using a multinomial distribution, n | p ~ Multinomial(p_U_, p_D_, p_C_). We imposed a Dirichlet prior on the vector of proportions, p | a, w ~ Dirichlet(a, a, wa) with w ≥ 1; this prior reflects the “null hypothesis” that the same number of genes should change expression levels in each direction (p_U_ = p_D_) and that most genes do not change (p_C_ >> max(p_U_, p_D_)). We computed the posterior probability that more genes have increased expression level (up category) compared to a decreased expression level (down category), using both an uninformative uniform prior (a = w = 1) and a strongly conservative informative prior that very few genes change expression (a = 1, w = 100). See Table S5 for the results for individual cell lines; see Table S6 for the results after pooling cell lines by type.
