# Supplementary material for: Genes suppressed by DNA methylation in non-small cell lung cancer reveal the epigenetics of epithelial–mesenchymal transition
Source: BMC Genomics. 2014 Dec 8;15(1):1079. doi: 10.1186/1471-2164-15-1079 (PMC4298954; doi:10.1186/1471-2164-15-1079)
Supplement: Supplementary file 13 — Additional file 13: Table S6: Posterior probability that more genes increase (rather than decrease) expression after treatment with 5AZA, by type of cell line, using either an uninformative prior or a conservative prior that assumes most genes do not change expression. (DOCX 18 KB) [file 12864_2014_6772_MOESM13_ESM.docx]

**Table S6.** Posterior probability that more genes increase (rather than decrease) expression after treatment with 5AZA, by type of cell line, using either an uninformative prior or a conservative prior that assumes most genes do not change expression.

| **Cell Lines** | **Gene Set** | **Uninformative Prior** | **Conservative Prior** |
| --- | --- | --- | --- |
| M-type | M-GRM | **1.0000** | **1.0000** |
| E-type | M-GRM | 0.0879 | 0.0824 |
| M-type | E-GRM | 0.1904 | 0.1980 |
| E-type | E-GRM | **0.9999** | **0.9997** |

5AZA = 5-azacytidine (an inhibitor of DNA methylation); M = mesenchymal-like; GRM = gene regulatory module; E = epithelial-like

Posterior probabilities greater than 95% are shown in boldface.
