# Supplementary material for: Genes suppressed by DNA methylation in non-small cell lung cancer reveal the epigenetics of epithelial–mesenchymal transition
Source: BMC Genomics. 2014 Dec 8;15(1):1079. doi: 10.1186/1471-2164-15-1079 (PMC4298954; doi:10.1186/1471-2164-15-1079)
Supplement: Supplementary file 14 — Additional file 14: Table S7: Posterior probability that more genes increase (rather than decrease) expression in a cell line after treatment with 5AZA, using either an uninformative prior or a conservative prior that assumes most genes do not change expression. (DOCX 18 KB) [file 12864_2014_6772_MOESM14_ESM.docx]

**Table S5.** Posterior probability that more genes increase (rather than decrease) expression in a cell line after treatment with 5AZA, using either an uninformative prior or a conservative prior that assumes most genes do not change expression.

| **Cell Line** | **Gene Set** | **Uninformative Prior** | **Conservative Prior** |
| --- | --- | --- | --- |
| A549 (M-type) | M-GRM | **0.9546** | **0.9551** |
| H1299 (M-type) | M-GRM | **1.0000** | **1.0000** |
| H157 (M-type) | M-GRM | **1.0000** | **0.9999** |
| H460 (M-type) | M-GRM | **0.9924** | **0.9939** |
| H1819 (E-type) | M-GRM | 0.7458 | 0.7519 |
| H1993 (E-type) | M-GRM | 0.5021 | 0.4985 |
| H2347 (E-type) | M-GRM | 0.0400 | 0.0377 |
| A549 (M-type) | E-GRM | 0.9070 | 0.9113 |
| H1299 (M-type) | E-GRM | **0.9887** | **0.9894** |
| H157 (M-type) | E-GRM | 0.0002 | 0.0001 |
| H460 (M-type) | E-GRM | 0.2819 | 0.2895 |
| H1819 (E-type) | E-GRM | 0.3084 | 0.3128 |
| H1993 (E-type) | E-GRM | 0.8871 | 0.8908 |
| H2347 (E-type) | E-GRM | **0.9998** | **1.0000** |

5AZA = 5-azacytidine (an inhibitor of DNA methylation); M = mesenchymal-like; GRM = gene regulatory module; E = epithelial-like

Posterior probabilities greater than 95% are shown in boldface.
